# Enzyme-Immobilized Microfluidic Process Reactors

**DOI:** 10.3390/molecules16076041

**Published:** 2011-07-19

**Authors:** Yuya Asanomi, Hiroshi Yamaguchi, Masaya Miyazaki, Hideaki Maeda

**Affiliations:** 1 Measurement Solution Research Center, National Institute of Advanced Industrial Science and Technology (AIST), 807-1 Shuku, Tosu, Saga 841-0052, Japan; 2 Liberal Arts Education Center, Aso Campus, Tokai University, Minami-aso, Aso, Kumamoto 869-1404, Japan; 3 Department of Molecular and Material Sciences, Interdisciplinary Graduate School of Engineering Science, Kyushu University, 6-1 Kasuga-koen, Kasuga, Fukuoka 816-8580, Japan; 4 Department of Advanced Technology Fusion, Graduate School of Science and Engineering, Saga University, 1 Honjo, Saga 840-8502, Japan

**Keywords:** microfluidic reactor, microreactor, immobilization, bioconversion

## Abstract

Microreaction technology, which is an interdisciplinary science and engineering area, has been the focus of different fields of research in the past few years. Several microreactors have been developed. Enzymes are a type of catalyst, which are useful in the production of substance in an environmentally friendly way, and they also have high potential for analytical applications. However, not many enzymatic processes have been commercialized, because of problems in stability of the enzymes, cost, and efficiency of the reactions. Thus, there have been demands for innovation in process engineering, particularly for enzymatic reactions, and microreaction devices represent important tools for the development of enzyme processes. In this review, we summarize the recent advances of microchannel reaction technologies especially for enzyme immobilized microreactors. We discuss the manufacturing process of microreaction devices and the advantages of microreactors compared to conventional reaction devices. Fundamental techniques for enzyme immobilized microreactors and important applications of this multidisciplinary technology are also included in our topics.

## 1. Introduction

Microfluidic reaction devices, which can be prepared by microfabrication techniques, or by assembly and modification of microcapillaries, constitute reaction apparatus with small dimensions, large surface to volume ratios and well defined reaction times [1,2,3]. These systems take advantage of microfluidics or nanofluidics that enables use of micro- or nanoliter volumes of reactant solutions and offer the advantages of high efficiency and repeatability. Therefore, microchannel reaction systems are expected to be the new and promising technology in the fields of chemistry, chemical engineering and biotechnology [4,5,6,7,8,9,10]. They offer several advantages over traditional technologies in performing chemical reactions. The key advantages of microsystems include rapid heat exchange and rapid mass transfer that cannot be achieved by the conventional batch system. Unlike macro scale solutions, streams of solutions in a microfluidic system mainly form laminar flow which allows strict control of reaction conditions and time. In addition, microchannel reaction systems provide large surface and interface areas, which are advantageous for many chemical processes such as extractions and catalytic reactions. Several chemical reaction devices have been reported to demonstrate potential applications [4,5,6,7,8,9,10]. Moreover, many potential applications for miniaturized synthetic reactors require only small volumes of catalysts. 

Enzymatic conversions have recently attracted considerable attention because of their environmentally-friendly nature. Several enzyme processes have been developed; however, improvement of the entire process is still required to obtain the benefits that can be derived from their use and for them to become a common or standard technology [11,12]. Reaction engineering might provide solutions to develop enzyme reaction processes at the commercial level [13], and microreaction engineering is one candidate for such technology. Therefore, several techniques have been developed, either in solution phase or by immobilizing enzymes, to realize enzyme microreaction processes [10,14]. In this review, we summarize recent advances in microchannel reaction technologies, focusing especially on enzyme-immobilized microreactors. We discuss the manufacturing process of microreaction devices and the advantages of microfluidic systems compared to conventional reaction devices. Fundamental techniques for enzyme-immobilized microreactors and important applications of this multidisciplinary technology are also presented.

## 2. Microreactor Fundamentals 

For the beginner unfamiliar with microfluidic reaction techniques, we would like to start our review with a brief introduction to microreactors. Microfluidic reactions occur in a small space within a reaction apparatus. Continuous-flow systems are mainly employed, and in most cases mechanical pumping, commonly by syringe pumping, or electroosmotic flow, which is the motion of ions in a solvent environment through very narrow channels, where an applied potential across the channels causes ion migration, are used as the driving force of the reaction systems. Microreaction devices developed so far can be classified into two types: chip-type microreactors and microcapillary devices. Chip-type microreactors which offer several advantages, including easy control of microfluidics, and integration of many processes into one reaction device. Chip-type microreactors have been mainly used for the development of bioanalytical devices. The manufacturing processes of such devices were adapted mainly from the microelectronics industry. Dry-or wet-etching processes have been used for creating channels on a silicone or glass plates. Polymer-based materials can be used for preparation of enzyme microreactors because most enzyme reactions are performed in aqueous solution, especially for bioanalytical use. Polydimethylsiloxane (PDMS), polymethylmethacrylate (PMMA), poly-carbonate, and Teflon were used for preparation of microreaction devices. These plates could be processed by photolithography, soft lithography, injection molding, embossing, and micromachining with lasers or microdrilling. The LIGA (Lithographic Galvanoforming Abforming) process which consists of a combination of lithography, electrochemical technology and molding, can also be used for the production of microreactors. 

In a microreactor, stable formation of laminar streams of different solutions is sometimes required, although some cases require better mixing by disrupting laminar streams. Methods for stabilizing multiple laminar flows and micromixing have been developed. Tokeshi *et al*. developed guide structures at the bottom of microchannels [15]. The structures were prepared by wet etching of a glass plate. Laminar streams of organic solvent and water in these microchannels were stabilized by these guide structures. Techniques for partial surface modification of microchannels were also developed. These techniques take advantage of the different surface properties. Organic solvents prefer hydrophobic regions, whereas aqueous solutions go to hydrophilic regions. Modification of glass by octadesylsilane was used to stabilize the flow of organic solvent and aqueous solutions [16]. In another study, a UV-sensitive self-assembled monolayer with fluorous chains was used for preparing partially-modified microchannel surfaces [17]. Our laboratory developed another method to stabilize solutions [18]. Microchannels were fabricated on both bottom and top plates. The microchannels of one of the two plates were coated with gold, then treated with alkanethiol to produce a hydrophobic surface. The resulting microreactor, which forms an upside-down laminar stream, was not only stabilized by interaction with surface, but also supported by gravity. Overall, such partial modification methods are useful to stabilize laminar streams under pressure below the critical value. Indeed, microfluidic phenomenon of laminar flow is one important aspect in the development of chip-type microreactors. 

Micromixers which enhance mixing of two or more different solutions in microspace have also been constructed. Rapid mixing in microfluidics is difficult to achieve because under laminar flow mixing of fluids is principally limited to diffusion through the interface. Several micromixers were developed by adding devices or materials in the microchannel, such as electrokinetical mixing [19] and microbeads [20]. Various types of micromixers which only require structured microchannels were also developed. These include chaotic mixers with oriented ridges at the bottom of microchannels [21], repeated dividing and merging of fluids with a two-way separated serpentine flow path [22], zig-zag microchannels [23], and simple convergence to 32 layers with two solutions divided into 16 microchannels [24]. Detail of micromixing can be found in recent reviews [25]. Still, design and fabrication of highly efficient micromixers for effective functioning of microfluidic devices are desired research topics. 

The other type of microreaction device consists of microcapillaries. This is the simplest method which does not require any control of microfluidics, rather, it uses a microchannel as the reaction space. The major advantage of this type of microreactors is in scaling up process which can be achieved by simply bundling more microcapillaries. Gas or liquid chromatography parts are chiefly used to prepare this type of microreactors. Capillary type microreactors are mainly used to develop manufacturing processes, especially catalytic reactions, to take advantage of the large surface area. The users should select the type of microreactor depending on the feature of reaction which they want to perform in a microreactor.

## 3. Fundamental Techniques for Enzyme Immobilized Microreactor

### 3.1. Enzyme-Immobilization within Microchannels

In the development of enzyme processes, the use of immobilized enzymes is preferable. Several methods have been used to immobilize enzymes on supports in conventional reaction apparatus, and these techniques have also been applied to immobilize enzyme within a microspace (Table 1, Table 2 and Table 3).

In batchwise reactors, immobilization of enzymes on beads or monoliths has been used for separation and recycling of enzymes. This approach has also been applied to microreaction systems. Microreactors with enzymes immobilized on glass beads have been prepared by simply filling the reaction chamber with enzyme-immobilized particles. Such a device was used for the determination of xanthine using chemiluminescent detection [26]. Crooks and co-workers developed advanced analytical microreactors using enzyme-immobilized microbead-mixing [20], and efficiently performed multistep enzyme reactions using glucose oxidase- and horseradish peroxidase-immobilized polystyrene. In addition, immobilization of enzyme on Ni-NTA-agarose bead has also been reported [27]. This immobilized enzyme is less denaturated because binding of the enzyme is achieved using a His-tag. This method was applied to immobilize bacterial P_450_ [27]. A similar approach was applied to immobilize enzymes onto a Merrifield resin [28]. A tyrosine-based Ni-NTA linker was created on the surface of the resin to immobilize His-tagged enzymes. This matrix was loaded into a microstructured channel of a PASSflow^TM^ system. Synthesis of (*R*)-benzoin, (*R*)-2-hydroxy-1-phenylpropan-1-one, and 6-*O*-acetyl-d-glucal were performed using this system. Magnetic beads were also used for enzyme immobilization within the microchannel. Glucose oxidase was immobilized within a Teflon tube by placing a magnet [29]. The enzyme-immobilized magnetic particles were stable and active for more than eight months. This approach was also applied for the preparation of a protease-immobilized microreactor for proteomic analysis [30]. A similar technique was used for preparation of enzyme-immobilized microfluidic reactors.

Monolithic microreactors can be prepared using several methods. A trypsin-immobilized microreactor was prepared by molding a porous polymer monolith, prepared from 2-vinyl-4,4-dimethylazlactone, ethylene dimethacrylate, and acrylamide or 2-hydroxyethyl methacrylate, with an enzyme, in microchannels [31]. This microreactor was used for mapping protein digested fragments. Preparation of a microreactor by filling a silica monolith made from tetraethoxysilane with an enzyme and entrapping it within a microchannel was also developed. Trypsin-encapsulated monolith was fabricated *in situ* on a PMMA microchip to produce an integrated bioreactor that can perform enzymatic digestion, electrophoretic separation and detection in one chip [32]. Another example is a protease-P-including monolith prepared from a mixture of tetramethoxysilane and methyltrimethoxysilane (1:4), used to fill in PEEK [poly(ether ether ketone)] microcapillary to produce a microreaction system [33]. Aluminum oxide powder can be used as a solid support. Horseradish peroxidase was immobilized on aluminium oxide with 3-aminopropylsilane, and then placed within the microdevice [34]. This method takes advantage of the porous nature of ceramic microstructures. Overall, preparation of immobilized enzymes with powdered materials or monoliths is significantly easier; however it is unfavorable in large scale processing because of increasing pressure.

### 3.2. Immobilization of Enzyme on Microchannel Surface

Methods for enzyme immobilization on the microchannel surface have also been developed because they can take advantage of the larger surface area of microreaction systems without pressure increases. Physical immobilization is an easy way to immobilize molecules. In microchannel systems, a biotin-avidin system has mainly been used to immobilize enzymes. The biotinylated polylysine was physically immobilized on a glass surface to immobilize streptavidin-conjugated alkaline phosphatase [40]. This microreactor was used for rapid determination of enzyme kinetics. Biotinylated lipid bilayer [41] and partial biotinylation by photo patterning on fibrinogen [42] were also used for immobilization. However, these methods are not suitable for long-term use because of their instability. Also, applications are limited to streptavidin-conjugated enzymes.

The introduction of a functional group on the microchannel surface was used for covalent cross-linking. A trypsin-immobilized microreactor was prepared by modification with 3-aminopropylsilane and glutaraldehyde using the classical method [43]. Although this immobilization method is easy, fabrication of complex microstructures is required to achieve high performance. Our group developed a modified sol-gel technique to form nanostructures on a silica microchannel surface [44]. This method modifies the microchannel surface with polymerized copolymer of 3-aminopropylsilane/methylsilane. Using this method, increased surface area was obtained. At least 10 times more enzymes can be immobilized on these nanostructures by covalent cross-linking through amide-bond formation, disulfide or His-tag, by modifying succinate spacer, compared with single layer immobilization [45,46,47]. A microreactor with immobilized cucumisin on the nanostructured surface could process substrate 15 times faster than the corresponding batchwise reaction [46]. 

**Figure 1:**
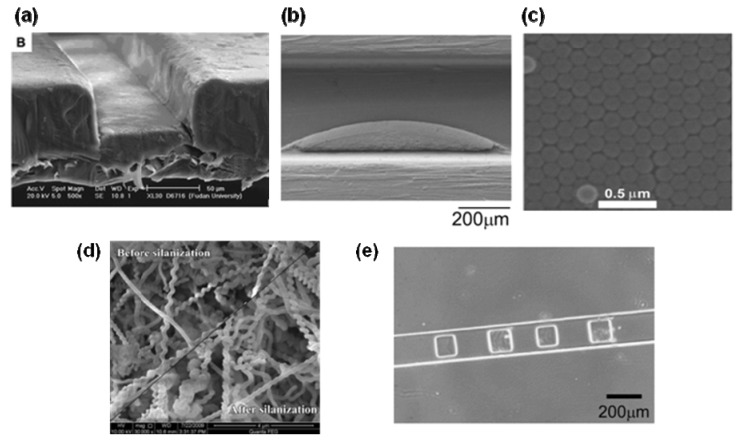

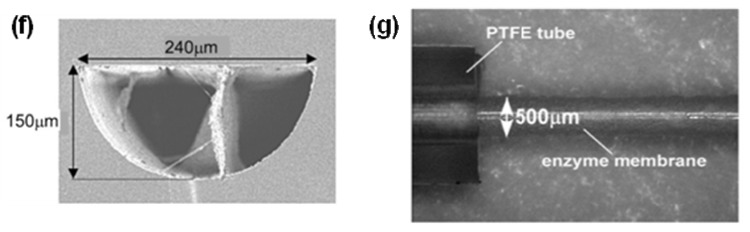
Images of surface modification and membrane formation techniques for micro enzyme reactor. Modified surface obtained by functionalized microstructure fabricated from layer-by-layer nanozeolite-assembled network (**a**), silicone rubber (**b**), nanoparticle arrangement (**c**), SiO_2_ nanospring structures (**d**), and hydrogel formation (**e**). Membrane formed within the microchannel can also be used as support for enzyme immobilization. Nylon membrane formed at liquid-liquid interface (**f**), or membrane of cross-linking enzyme aggregate formed at microchannel surface (**g**) was used for immobilization. These images were reproduced with permission from references [50,51,53,55,57,58,61].

Similar surface modification methods employing sol-gel techniques were also developed [48]. A PMMA surface was modified with a copolymer of butyl methacrylate/γ-(methylacryloxy)-propyltrimethoxysilicane and silica-sol-gel to immobilize enzymes. Using this method, a trypsin-immobilized microreactor was developed. In addition, a trypsin-encapsulated titania and alumina gel matrix was immobilized through SiOH group formed on a PDMS surface by plasma oxidation [49]. Using this device, digestion time was significantly shortened (ca. 2 s) and the application for high-throughput protein identification was realized. Ji *et al*. developed the layer-by-layer nanozeolite-assembled network to immobilize enzymes in the porous structure formed within zeolite (Figure 1a) [50]. Alternatively, silicone rubber material was used for the preparation of functional nanostructure on the microchannel surface (Figure 1b) [51]. 

The structure was prepared by micromold fabrication using vinyl-group-containing PDMS and silicic acid, and enzyme immobilization by cross-linking with glutaraldehyde. Using this procedure, a microstructured enzyme reactor with immobilized thermophilic β-glycosidase capable of performing hydrolysis at 80 °C was created. A particle-arrangement technique was also applied for enzyme immobilization. Silica nanoparticles were immobilized onto the surface using slow evaporation of particle suspension filled-in microchannel (Figure 1c) [61]. The obtained microchannel was subjected to treatment with 3-aminopropyltriethoxysilane, and immobilization of enzyme was achieved by covalent cross-linking through the amino groups. Although physical stability needs to be improved, a lipase-immobilized microreactor prepared by this method showed 1.5 times faster kinetics than that of microreactor obtained by sol-gel surface modification [52]. This result showed good correlation with the surface area; particle arrangement has approximately 1.5 times larger surface area and could immobilize more enzymes. A SiO_2_ nanospring structure formed by chemical vapor deposition was also used as immobilization supports. (Figure 1d) [53]. Photochemistry has been applied to enable selective immobilization of enzymes on the microchannel surface [54]. In the procedure, vinyl azlactone was photografted onto a PEG-coated polymer surface as a reactive monomer and the enzymes were immobilized through their amino groups. This approach was applied for immobilization of horseradish peroxidase. Another approach for efficient enzyme immobilization is polymer coating. Poly(ethylene glycol)based-hydrogels which incorporate alkaline phosphatase was prepared within a microchannel by exposure to UV light (Figure 1e) [55]. This method was also applied to immobilize urease and different enzymes on microchannel surfaces. Overall, these techniques need expensive equipments and/or specialized fabrication skills.

### 3.3. Membrane-Formation

Enzymes can be immobilized on a membrane within the microchannel. A porous poly(vinylidene fluoride) membranes embedded within microchannel can be used for enzyme immobilization. Preparation of a miniaturized membrane reactor by absorption of enzymes onto the membrane has been reported [56].

Hisamoto *et al*. demonstrated that a nylon-membrane formation at the interface of two solutions formed in a microchannel (Figure 1f). Peroxidase was immobilized on this membrane which was then used as a chemicofunctional membrane [57]; however, immobilization of the membrane is technically difficult, and application of this method is limited because the nylon-membrane is unstable in organic solvents. 

We have developed a technique that forms an enzyme-immobilizing membrane on the microchannel surface [58]. This is a modification of cross-linked enzyme aggregate (CLEA) formation, which is used in batchwise organic synthesis [62]. Simple loading of the enzyme solution and a mixture of glutaraldehyde and paraformaldehyde into the microchannel forms a CLEA membrane on the microchannel wall (Figure 1g). The resulting microreactor can be used for prolonged periods (>40 days), and shows excellent stability against organic solvents. Taking into account these advantages, this method is considered ideal for the development of an enzymatic reactor tailored for specific applications. However, this method requires amino groups for immobilization, and is difficult to apply to acidic enzymes with few amino groups on their surface. The application of the approach developed in our laboratory was expanded by adding poly-Lys to aid in membrane formation of acidic proteins [59]. By this method, almost all enzymes, including highly acidic proteins, can form cross-linked aggregates. We applied this technique for the preparation of enzyme microreactors, and demonstrated immobilization of several acidic enzymes by this method [59]. Our results indicate that almost all enzymes can be immobilized onto the microchannel surface by our method, and our approach is a robust way of enzyme-immobilized microreactor development.

## 4. Enzyme-Immobilized Microfluidic Reactor Processes 

### 4.1. Hydrolysis and Esterification

Applications of enzyme-immobilized microreactors for processing for several important reactions in the synthetic organic chemistry field have been reported. As shown in Table 4, esterification and hydrolysis reactions are important processes in the industry that have also been performed in a microchannel system. Lipase-immobilized microreactors were prepared using a ceramic microreactor and glass microcapillaries [45], wherein hydrolysis of the ester was conducted. Both microreactors showed 1.5 times better yield than the batchwise reaction using the same volume/enzyme ratios. This could have resulted from an increase in contact due to the larger surface area of the microchannel systems. Hydrolysis of triglyceride using a lipase-immobilized microreactor was also reported. The reaction yield was 10 times higher than that of the corresponding batchwise reaction [63]. Hydrolysis of vegetable oil to produce monoacyl glycerol was performed in an immobilized lipase microreactor [64]. Almost complete conversion was enabled by the microreaction system. Not only hydrolysis, but esterifications were also performed in microfluidic format. A microreaction using immobilized Novozym-435^TM^ was also reported, where esterification of diglycerol with lauric acid was performed [65]. 

Esterases are also used as catalysts to produce esters and their hydrolysis products. Dräger *et al*. reported a regioselective hydrolysis reaction by *p*-nitrobenzyl esterase immobilized onto Ni-NTA agarose beads entrapped within microchannels [28]. Although an 80% yield was obtained with the microreactor, trace by-products were also detected. Proteases and aminoacylases are important tools for the preparation of chiral compounds. A monolithic microreactor tethered protease P was applied for bioconversion processes. Transesterification of (*S*)-(-)-glycidol and vinyl *n*-butyrate was performed using this microreaction device [33], but the conversion depended on the amount of immobilized enzymes.

Similarly, they separated the racemic product which was obtained by reaction in an entrapped lipase microreactor, by connecting a chiral column sequentially to the microreactor [35]. We developed a novel integrated microreaction system which combined an enzyme microreactor and a solvent extractor. The enzyme-immobilized microreactor was prepared by the membrane-formation technique using α-aminoacylase with poly-Lys [59]. This microreactor was connected with a microextractor which has a partially modified microchannel [18]. Using this microreaction system, optical resolution of d/l-phenylalanine analogs was performed. The d-phenylalanine analogs were obtained efficiently with high optical purity [60].

### 4.2. C-C Bond Formation, Condensation and Addition

Processing with C-C bond formation, condensation and addition reactions performed in a microchannel system are shown in Table 5.

Hydroxylation of macrolides in a microreactor was reported [27]. PikC Hydroxylase was immobilized on Ni-NTA agarose beads, and then filled into the microchannel. This microreactor was used for hydroxylation to produce methymycin and neomethymycin, and over 90% conversion was achieved at a flow rate of 70 nL/min. Such high efficiency might have resulted from the shorter residence time, which is preferable for enzymes with inherent stability. Similar immobilization technique was applied for the synthesis of (*R*)-benzoin using benzaldehyde lyase [28]. His-tagged protein was directly immobilized within the microstructured PASSflow reaction system through tyrosine-based Ni-NTA system. This reversible immobilization technique was also used for transketolase, which catalyses the synthesis of l-erythrulose [66]. Its productivity was unchanged over five regeneration cycles. Schwarz *et al*. reported transglycosilation using cellobiose and glycerol to produce β-glycosylglycerol [67]. The enzyme was immobilized onto the γ-alumina layer formed within a microchannel. However, the resulting microreactor showed almost similar conversion characteristics as the batchwise reaction.

### 4.3. Oxidation and Reduction

The application of enzyme-immobilized microreactors for oxidation and reduction was also reported (Table 6). Although the uses of oxidation reactions with the enzyme-immobilization techniques were mainly for analytical use, the oxidation of phenols with a horseradish peroxidase-immobilized microreactor was recently reported [68].

We demonstrated the reduction of pyruvic acid to produce l-lactic acid by l-lactic dehydrogenase immobilized on microchannel surface through Ni-NTA group formed by sol-gel technique [47]. By this method, crude enzyme extract from bacterial lysed solution could be used for immobilization without further purification. Also, reversible immobilization was enabled to regenerate the microreactor upon enzyme denaturation. This reactor showed higher conversion rates than that of the batchwise reaction; however regeneration of co-enzyme still remains a major problem in this case. Yoon *et al*. reported an electrochemical microreactor for regeneration of coenzymes [69]. However, they used solution-phase reactions using enzyme solutions. Integration of enzyme immobilization techniques with this microreactor for co-enzyme regeneration might solve this problem.

### 4.4. Miscellaneous Reactions

Enzymatic polymerization has been performed in microfluidic format. Entrapped Novozym-435^TM^ was used for ring-opening polymerization of ε-caprolactone to produce polycaprolactone [70]. The microreactor showed improved reaction rates, higher than those observed in the batchwise reaction. Glycosyltransferase-entrapped monolith was used for the preparation of oligosaccharides from monosaccharides [37]. The immobilized enzyme was stable and the resulting microreactor exhibited good reproducibility.

The application of enzyme-immobilized microreactors for multistep synthesis was also demonstrated [71]. Three separate microfluidic devices, which possesed metallic zinc, silica-immobilized hydroxyaminobenzene mutase, and silica-immobilized peroxidase within a microchannel, were prepared and connected sequentially. These devices were used for combinatorial synthesis of 2-aminophenoxyazin-3-ones. These results open the door for the application of micro bioreactors for the enzymatic synthesis of bioactive natural products.

## 5. Conclusions

Microchannel devices can be useful in imitating biological reaction apparatus, such as cellular surfaces and vascular systems, by providing the advantages of limited space and laminar flow compared with the conventional reaction apparatus. The quest for microreaction technologies will lead to better process intensification and efficient analytical methods. Increasingly, new findings are being achieved in microfluidics. Further investigation on microfluidics could provide novel mechanisms not observed in conventional systems, and better understanding of fluidics in microchannels might enable new reaction pathways not possible with conventional systems. 

The strong advantages offered by microreaction devices are useful, particularly in the development of microreaction systems for commercial purposes. Once a microreactor is optimized, it can be easily introduced into an industrial-scale plant. Parallel scale-out enables extension of reaction conditions optimized in a single reactor, and eliminates scale-up problems arising from conventional processes. Parallel operation of the same microreaction provides high throughput operation of different reagents at a single operation and serves as an excellent tool for combinatorial processing. Although several problems, such as connection, parallel control of fluid and reaction conditions, and monitoring, are common challenges, the benefits offered by microreaction technology accelerate the development of enzyme reaction devices. 

As described here, few enzymes have been applied for microreaction process development, and not many patents describing the construction of micro enzyme reactors are published. These facts are an indication that the field is still in its initial stages. Efforts directed to the development, optimization and application of micro enzyme reactors will open a new era for biochemical processing in the synthetic organic chemistry field.

## Figures and Tables

**Table 1:** Enzyme-immobilization within microchannel reactors by particle entrapment techniques.

Media	Immobilization method	Enzyme	Advantage and disadvantage	Ref.
Glass	Cross-linking (3-aminopropylsilane/ glutaraldehyde)	Xantin oxidase Horseradish peroxidase	Ease in preparation Enable multistep reaction Limited number of enzymes are applicable due to denaturation Pressure gain	[26]
Polystyrene	Biotin-Avidin (Avidin-coated beads were used)	Horseradish peroxidase	Ease in preparation Enable multistep reaction Biotin-label is required Pressure gain	[20]
Agarose	Complex formation (Ni-NTA and His-tag)	Horseradish peroxidase	Ease in preparation Applicable for engineered enzymes Higher pressure by increasing flow rate and particles may be crushed	[27]
Polystyrene	Complex formation (Ni-NTA and His-tag)	Glucose oxidase	Ease in preparation Applicable for engineered enzymes Higher pressure by increasing flow rate and particles may be crushed	[28]
Magnetic bead	Cross-linking (3-aminopropylsilane/ glutaraldehyde)	Bacterial P450	Preparation is easy Enzyme can be immobilized on any place by placing a magnet Amount of enzyme particle is limited because of plugging	[29,30]
Polymer monolith	Entrapment(2-vinyl-4,4- dimethylazlactone, ethylenedimethacrylate, 2-hydroxyethyl methacrylate, acrylamide)	Benzaldehyde liase	Stabilization of enzyme structure and activity Requirement of skill in preparation Denaturation during entrapment process	[31]
Silica monolith	Entrapment within porous silica	*p*-Nitrobenzyl esterase	Stabilization of enzyme structure and activity Compatibility in organic solvent Requirement of skill in preparation Denaturation possible during entrapment process	[32,33,35]
Aluminium oxide	Cross-linking (3-aminopropylsilane/ glutaraldehyde)	Glucose oxidase	Large surface area due to porous nature Applicable for heterogeneous reactions Complicated preparation Not applicable for large-scale processing	[34]
Porous polymer monolith	Multistep photografting	Trypsin LysC	Eliminate nonspecific adsorption of proteins and peptides	[36]
CIM-disk epoxy monolith	Entrapment within monolith	Glycosyltransferases	CIM^®^ Epoxy Disk Monolithic Column is available for purchase	[37]
Caged mesoporous silica in Ca- alginate fiber	Entrapment within amine-modified mesoporous silica	Glucose oxidase	Reduced leakage and improved activity and stability of the immobilized enzyme	[38]
LTCC multilayer substrates	Cross-linking (Glyoxal-agarose gels)	β-galactosidase	Stable operation for 6 months	[39]

**Table 2:** Typical techniques for enzyme-immobilization on microchannel surfaces.

Media	Immobilization method	Enzyme	Advantage and disadvantage	Ref.
SiO_2_ surface	Physical adsorption of biotinylated poly-lysine /biotin-avidin	Alkaline phosphatase	Ease in preparation Requirement for avidin-conjugation Possible occurrence of detachment	[40]
PDMS (O_2_ Plasma treated)	Physical adsorption of lipid bilayer/biotin-avidin	Alkaline phosphatase	Enable immobilization of enzyme on plastic surface Possible occurrence of detachment Expensive reagents Requirement for avidin-conjugation	[41]
PDMS	Physical adsorption of fibrinogen/Photochemical reaction of Fluorescein- biotin	Alkaline phosphatase	Enable partial modification of microchannel Special equipment is required	[42]
Silicon	Cross-linking (3-aminopropylsilane/ glutaraldehyde)	Trypsin	Simple operation Difficulty in channel preparation Poor reproducibility	[43]
Fused silica (Sol-gel modified)	Cross-linking (3-aminopropylsilane/ glutaraldehyde)	Cucumisin Lipase l-Lactic dehydrogenase	Simple operation Immobilize ~10 times more enzymes than single layer immobilization and therefore, performs with higher reaction efficiency Several chemistry is available (amide, disulfide, His-tag) Needs several steps for immobilization Reproducibility strongly affected by characteristics of silica surface	[44,45,46,47]
PMMA	Cross-linking (Si-O bond between modified surface and silica monolith)	Trypsin	Stabilize enzyme under denaturation condition Complicated preparation method	[48]
PDMS (O_2_ Plasma treated)	Cross-linking (Si-O-Ti or Si-O-Al bond between titania or alumina monolith)	Trypsin	Stabilizes enzyme under denaturation condition Complicated preparation method	[49]
PET microchip	Entrapment within nanozeolite-assembled network	Trypsin	Large surface/volume network by layer-by-layer technique	[50]
Silicon rubber	Cross-linking (3-aminopropyltrieth-oxysilane and glutaraldehyde)	Thermophilic β- glycosidase	Reaction can be performed at 80 °C Complicated preparation method Reaction is slow because not much enzyme can be immobilized	[51]
Fused silica	Cross-linking between physically-immobilized Silica particle (3-aminopropylsilane/succinate)	Lipase	Much larger surface area (1.5 times greater than sol-gel modified surface) and higher efficiency Complicated preparation method Unstable withed physical force (bending etc.)	[52]
SiO_2_ nanospring	Disulfide bond	β-galactosidase	High solvent-accessible surface area permeability and mechanical stability Repeatability of re-immobilization was poor	[53]
Photopatterning onto PEG-grafted surface	Cross-linking by photo-patterned vinylazlactone	Horseradish peroxidase Glucose oxidase	Reduced non-specific absorption Sequentially multistep reaction could be achieved Requires special equipment	[54]
PDMS	Entrapment within hydrogel formed on surface	Alkaline phosphatase Urease	Quite fast reaction (90% conversion at 10 min reaction) Immobilization of multiple enzyme Complicated preparation method Not applicable for higher flow rate	[55]

**Table 3:** Enzyme-immobilization techniques on a membrane.

Media	Immobilization method	Enzyme	Advantage and disadvantage	Ref.
PDMS/Glass	Place PVDF membrane that adsorbs enzymes	Trypsin	Easy preparation Less efficiency Possibility of leakage at higher flow rate	[56]
Glass	Covalent cross-linking with Nylon membrane formed at liquid-liquid interface (glutaraldehyde)	Horseradish peroxidase	Integration of membrane permeation and enzyme reaction Preparation of multiple membrane Complicated preparation method Unstable membrane at higher flow rat	[57]
PTFE	Enzyme-embedded membrane formation using glutaraldehyde/ paraformaldehyde	α-Chimotrypsin Trypsin α-Aminoacylase Other various enzymes	Easy preparation Durable (>40days) Applicable in organic solvents Almost all enzymes can be immobilized by adding poly-Lys	[58,59,60]

**Table 4:** The use of enzyme-immobilized microreactors for hydrolysis and esterification.

Immobilization technique	Enzyme	Reaction scheme	Results	Ref.
Surface modification of silica capillary by sol-gel technique/immobilized through amide bond formation using succinate linker	Lipase	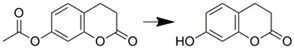	1.5 time better yield was obtained compared with batchwise reaction	[45]
Entrapment within folded-sheet mesoporous silicas	Lipase	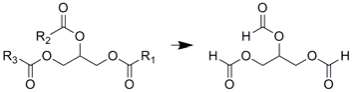	Reaction yield was 10 time higher than batchwise reaction	[63]
Covalently immobilized in silica micro structured fiber	Lipase	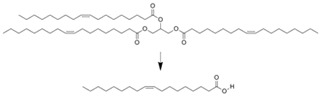	Almost complete conversion of a vegetable oil to monoacylglycerol	[64]
Entrapment of Novozym-435^TM^ within microchannel	Lipase	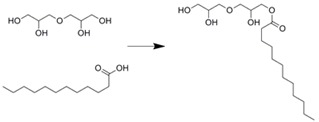	Much less of the reactant was required compared with the batchwise test	[65]
Ni-NTA agarose bead immobilization	*p*-Nitrobenzyl esterase	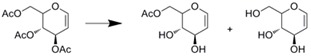	80% yields were obtained along with traces of byproduct	[28]
Silica monolith entrapped within microchannels	Protease P	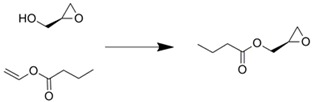	Conversion within microreactor was higher than that of the batchwise reaction at higher flow rates	[33]
Silica monolith entrapped within microchannels	Lipase	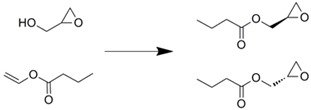	Optical resolution of products was achieved by connecting commercially available chiral column	[35]
Membrane formation with paraformaldehyde, glutaraldehyde, and poly-Lys	α-Amino-acylase	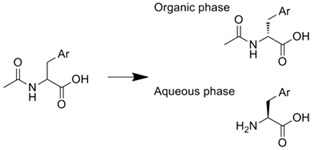	Optical resolution of d/l-amino acids were achieved by connecting to micro solvent extractor	[60]

**Table 5:** Processing with C-C bond formation, condensation and addition.

Immobilization technique	Enzyme	Reaction scheme	Results	Ref.
Ni-NTA agarose bead immobilization	PikC hydroxylase (Bacterial P450)	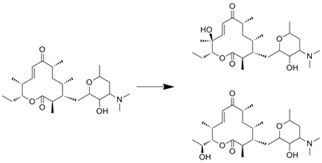	>90% conversion was obtained at 70nm/min	[27]
Ni-NTA agarose bead immobilization	Benzaldehyde liase	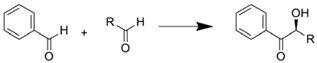	>90% yields were obtained	[28]
His_6_-tag affinity	Transketolase	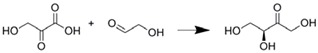	Productivity was unchanged over 5 cycles of regeneration	[66]
Covalently immobilized on layer of γ-aluminum oxide	Thermostable β-glycosidase CelB	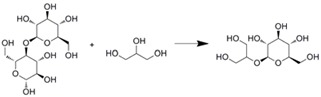	Similar conversion characteristics with batchwise stirred reactor	[67]

**Table 6:** Oxidation, reduction and miscellaneous reactions in enzyme-immobilized microreactor.

Immobilization technique	Enzyme	Reaction scheme	Results	Ref.
Covalently immobilized on gold patterned surface	Horseradish peroxidase	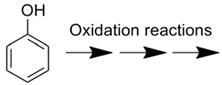	Conversion with self-assembled monolayer approach was 1.5 time higher than physical adsorption	[68]
Surface modification by sol-gel technique/Ni-NTA immobilization	l-Lactic dehydrogenase	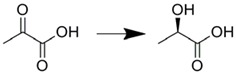	Crude enzyme can be used for immobilization Reversible immobilization was achieved by EDTA treatment Reaction was completed within 15 min	[47]
Entrapment of Novozym-435^TM^ within microchannel	Lipase	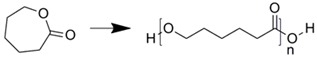	Apparent rate of reaction is at least an order higher than that observed for batch reactors	[70]
CIM-disk epoxy monolith	Glycosyl-transferases	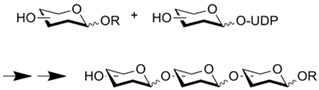	Immobilized enzyme is stable and exhibits good reproducibility	[37]
Entrapment of silica-immobilized enzymes within microchannel	Zinc Hydroxy-aminobenzene mutase Peroxidase	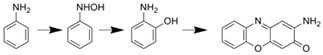	Used combinatorial synthesis of 2-aminophenoxyazin-3-one	[71]
